# Epigenetics and Autoimmune Thyroid Diseases

**DOI:** 10.3389/fendo.2017.00149

**Published:** 2017-06-29

**Authors:** Fabio Coppedè

**Affiliations:** ^1^Department of Translational Research and New Technologies in Medicine and Surgery, Section of Medical Genetics, University of Pisa, Pisa, Italy

**Keywords:** autoimmune thyroid diseases, Graves’ disease, Hashimoto’s thyroiditis, epigenetics, DNA methylation, non-coding RNAs, microRNA, histone tail modifications

## Abstract

Increasing evidence suggests that epigenetic modifications, including changes in DNA methylation, covalent modifications of histone tails, and gene silencing mediated by non-coding RNA molecules, play a substantial role in the pathogenesis of autoimmune disorders and might be seen as the result of environmental insults that trigger these conditions. Studies in cells and tissues of patients with autoimmune thyroid diseases (AITD), and particularly in Graves’ disease (GD) and Hashimoto’s thyroiditis (HT), are increasingly revealing altered epigenetic marks and resultant deregulation of gene expression levels, but the available data are still limited to be translated into the clinical settings. Particularly, genome-wide methylation and histone tail modification screenings are limited to a few studies in GD patients, and the diagnostic values of the observed epigenetic changes or their potential prognostic utility are still unclear. Similarly, data concerning microRNA expression in AITD patients are largely descriptive and not yet translated into the clinics. In addition, studies relating certain environmental exposures to specific epigenetic changes in AITD and studies evaluating the crosstalk between different epigenetic mechanisms are largely missing. In summary, despite that there is a clear evidence of epigenetic impairment in AITD, further research is required for a better understanding of the epigenetic networks involved in disease pathogenesis, thereby opening the way for potential diagnostic and prognostic tools, as well as for epigenetic interventions in the patients.

## Introduction

Epigenetics is an umbrella term referred to heritable and reversible marks, such as DNA methylation or covalent modifications of histone tails, that regulate the chromatin structure and switch genes “on” and “off” without changing the primary DNA sequence (1). In addition, several classes of non-coding RNAs, ranging from small to long molecules, play a substantial role in the epigenetic regulation of gene expression (2). Some studies performed at the end of the last century revealed that CD4+ T cells treated with 5-azacytidine, a substance that inhibits DNA methylation, respond to the presentation of self antigens and cause a lupus-like syndrome when injected in mice (3, 4), suggesting that epigenetic mechanisms, and particularly impaired DNA methylation, could be involved in autoimmune reactions (3, 4). A few years ago, we reviewed the literature for studies addressing epigenetic modifications in autoimmune diseases, most of them were focused on systemic lupus erythematosus or rheumatoid arthritis (RA), but increasing evidence was available for other autoimmune pathologies, including autoimmune thyroid diseases (AITD), a group of disorders characterized by loss of immunological self-tolerance (5). The major AITD are Graves’ disease (GD) and Hashimoto’s thyroiditis (HT), both organ-specific autoimmune diseases characterized by lymphocytic infiltration of the thyroid gland with accompanying evidence of humoral and cellular immune system activation and female preponderance (6). In GD, the autoimmune process results in the production of thyroid-stimulating antibodies leading to hyperthyroidism, whereas in HT the immune response is destructive, leading in most cases to hypothyroidism (7). Genetic predisposition and environmental factors, such as infection, chemicals, and nutrition, play a role in the pathogenic process of autoimmunity (8). Recent studies have clearly demonstrated a significant increased risk of other autoimmune diseases in patients with AITD and there is evidence of genetic factors that influence the association of different autoimmune disorders (9). In this regard, the investigation of the genetic risk factors for AITD has revealed that some genes are unique for GD or HT, while others are common to both diseases or to AITD and other autoimmune diseases (10). Increasing evidence suggests that epigenetic modifications may be seen to bridge the gap between genetics and the environment (10, 11), so that epigenetic modifications of autoimmune-related genes, resulting from environmental exposure, are increasingly recognized to play a pivotal role in autoimmunity (5). This article critically discusses the most recent evidence of epigenetic modifications in AITD.

## DNA Methylation in AITD

DNA methylation consists of the addition of a methyl group to the DNA, mediated by enzymes called DNA methyltransferases (DNMTs). The best-characterized DNA methylation process is the addition of a methyl group to cytosine in a CpG dinucleotide context, forming 5-methylcytosine (5-mC). When the promoter region of a gene is methylated, the expression of that gene is repressed because methyl-CpG-binding domain (MBD) proteins recognize and bind to the methylated DNA and, in turn, recruit other epigenetic factors to enhance chromatin remodeling and transcriptional repression (12–14). DNA methylation is a physiological mechanism required for several cellular processes, including genomic imprinting, embryonic development, cell differentiation, X chromosome inactivation, repression of repetitive elements, and maintenance of the cellular identity (1).

### Skewed X Chromosome Inactivation (XCI) in AITD

Many, but not all, autoimmune diseases are more common in females than in males, with reported ratios ranging from 10:1 to 3:1 (15). A possible role of skewed XCI, mediated by epigenetic mechanisms, has been suggested in the etiology of AITD (16), RA (17), and scleroderma (18) to partially explain the female preponderance. The X chromosome contains several immune-related genes, including CD40 ligand (*CD40L*), forkhead box P3 (*FOXP3*), and toll-like receptor 7 (*TLR7*), and one of the two X chromosomes in each female cell is randomly inactivated by methylation to balance gene expression levels between males, that possess only one X chromosome, and females who have two copies of the X chromosome (19). In some females, however, this inactivation can predominantly occur to either the maternal or paternal X chromosome, and this phenomenon is referred to as skewed XCI (19). Concerning AITD, studies performed over the last two decades have addressed the link between skewed XCI and AITD risk (16, 17, 20–23). A meta-analysis of those studies confirmed significant skewing of XCI with GD and HT (23), and studies on twins revealed that skewed XCI may be causally associated with clinically overt AITD, but not with the presence of thyroid autoantibodies in euthyroid subjects (7). A more recent study in AITD patients revealed that the proportion of skewed XCI was not significantly different with respect to control subjects, but was higher in patients with intractable GD than in those with GD in remission, and in patients with severe HT than in those with mild HT, suggesting that skewed XCI is likely related to the prognosis of AITD, rather than to their development (19).

### Polymorphisms of Genes Involved in DNA Methylation and AITD Risk

DNA methylation depends on the cellular availability of dietary folates and related B-group vitamins, all required for the production of S-adenosylmethionine, the intracellular donor compound of methyl groups (24). Several investigators provided indirect evidence of impaired DNA methylation in AITD by addressing the role of genes involved in folate metabolism and DNA methylation reactions as genetic risk factors for AITD. Particularly, those studies investigated polymorphisms in *DNMT* genes or in methylenetetrahydrofolate reductase (*MTHFR*) and methionine synthase reductase (*MTRR*) genes, the two latter coding for folate-metabolizing enzymes (25, 26). rs1801133 in *MTHFR* was associated with reduced GD risk in women (25), while rs2228612 in *DNMT1* was linked to DNA hypomethylation and with the intractability of GD and rs1801394 in *MTRR* with the severity of HT (26). A more recent study addressed the contribution of *DNMT* gene polymorphisms in a large cohort of AITD patients composed by a total of 685 GD patients, 353 HT patients, and 909 healthy controls, revealing that both rs2424913 in *DNMT3B* and rs2228611 in *DNMT1* were associated with AITD susceptibility (27). Interestingly, *DNMT* gene polymorphisms have been associated with other autoimmune disorders, for example *DNMT3B* polymorphisms were linked to increased risk of oral lichen planus (28), with the progression of joint destruction in RA (29), and with increased risk of thymoma in patients with myasthenia gravis (30). Collectively those studies suggest that variants in *DNMT* genes might account for a shared susceptibility to various autoimmune disorders.

### Evidence of Impaired DNA Methylation in AITD

More direct evidence of impaired DNA methylation in AITD came from recent epigenetic screenings in blood samples, lymphocytes, and thyrocytes from the patients (Table 1). A genome-wide screening in peripheral blood cells of three GD patients and three age- and gender-matched controls revealed 82 hypermethylated and 103 hypomethylated genes in GD patients (31). Among them, the authors identified some candidate genes already associated to GD or other autoimmune diseases, such as the immunoregulatory factor *ADRB2* (hypermethylated), *ICAM1* (hypomethylated) coding for a glycoprotein of cell surface named intercellular adhesion molecule 1, *B3GNT2* (hypermethylated) involved in the regulation of lymphocyte activity, and others (31). Besides, the transcription of DNMT1 and MECP2 (a MBD protein) at the messenger RNA (mRNA) level was significantly decreased in GD patients compared with normal controls (31). Another genome-wide analysis of DNA methylation was performed in CD4+ and CD8+ T cells of 38 GD patients and 31 matched controls. The study revealed 365 and 3,322 differentially methylated CpG sites in CD4+ and CD8+ T cells, respectively (32). Among the hypermethylated CpG sites, the authors found enrichment of genes involved in T cell signaling (*CD247, LCK, ZAP70, CD3D, CD3E, CD3G, CTLA4*, and *CD8A*) and decreased expression of CD3 gene family members (32). Furthermore, the authors observed hypermethylation of the first intron of the thyroid-stimulating hormone receptor (*TSHR*) gene, a gene that contains several GD-associated polymorphisms (32). A more recent study revealed aberrant DNA methylation of the *ICAM1* gene promoter, associated with increased gene expression, in the thyrocytes of 35 AITD patients with respect to 35 sex- and age-matched controls (33).

**Table 1:** Epigenetic studies in patients with AITD.

Endpoint	Tissue	Disease	Findings	Reference
DNA methylation	PBMC	GD	Genome-wide screening revealed 82 hypermethylated and 103 hypomethylated genes	(31)

DNA methylation	CD4+ and CD8+ T cells	GD	Genome-wide screening revealed 365 and 3,322 differentially methylated sites in CD4+ and CD8+ T cells, respectively	(32)

DNA methylation	Thyroid gland	AITD	Impaired methylation and increased expression of the *ICAM1* gene	(33)

Histone tail modifications	PBMC	GD	Global reduction of histone 4 acetylation	(36)

Histone tail modifications	CD4+ and CD8+ T cells	GD	Reduction of histone 3 lysine 4 trimethylation (H3K4me3) and histone 3 lysine 27 acetylation (H3K27ac)	(32)

MicroRNA (miRNA) expression	PBMC	GD	No expression of miR-154*, miR-376b, and miR-431*in early disease stages	(39)

MiRNA expression	Serum	HT	Increased levels of miR-22, miR-375, and miR-451	(40)

MiRNA expression	Serum	GD	Increased levels of miR-16, miR-22, miR-375, and miR-451	(36)

MiRNA expression	CD4+ and CD8+ T cells	HT and GD	Differential expression of miR-200a and miR-155	(40)

MiRNA expression	Serum	GD	Correlation between circulating levels of miR-155 and miR-146a and Grave’s ophtalmopathy	(42, 43)

MiRNA expression	Plasma and CD4+ T cells	GD	Upregulation of Bcl-6 and downregulation of miR-346	(44)

MiRNA expression	PBMC and thyroid gland	HT	Downregulated miR-125a-3p expression resulting in upregulation of interleukin-23 receptor levels	(45)

MiRNA expression	PBMC	HT	Increased let-7e expression regulates interleukin 10 expression	(46)

MiRNA expression	Thyroid gland	HT	Increased miR-142-5p expression regulates claudin-1 expression	(47)

MiRNA expression	Thyroid gland	GD	Altered expression of 23 miRNAs with resulting deregulated expression of more than 2,000 messenger RNAs	(48)

*AITD, autoimmune thyroid diseases; GD, Graves’ disease; HT, Hashimoto’s thyroiditis; PBMC, peripheral blood mononuclear cells*.

## Histone Tail Modifications in AITD

Several posttranslational modifications occur on the histone tails of nucleosomes and are associated with either open or condensed chromatin structure. Collectively those modifications are involved in the regulation of gene expression, as well as in DNA repair, replication, and recombination processes, and include acetylation, methylation, phosphorylation, ubiquitylation, sumoylation, and other covalent modifications that directly influence the overall chromatin structure or regulate the binding of effector molecules (34). Among them, acetylation and methylation on histone tail residues represent the two best-characterized epigenetic marks regulating the chromatin structure (35). Histone tail acetylation is mediated by histone acetyltransferases and results in an open chromatin structure that allows transcription (35, 36). Histone tail methylation of core histones H3 and H4 can be associated with either chromatin condensation or relaxation, due to the fact that several sites for methylation are present on each tail (35, 36).

Little is known about histone tail modifications in AITD (Table 1). A pilot study in peripheral blood mononuclear cells (PBMC) of GD patients revealed reduced global histone H4 acetylation levels coupled with increased levels of histone deacetylase proteins with respect to healthy controls (36). Furthermore, the previously described genome-wide DNA methylation analysis in CD4+ and CD8+ T cells of GD patients (32) revealed that the hypermethylation of genes involved in T cell signaling was accompanied by decreased levels of H3K4me3 (histone 3 lysine 4 trimethylation) and H3K27ac (histone 3 lysine 27 acetylation), both marks usually found in nucleosomes that flank active promoters (32). Collectively, those studies confirm that gene promoter methylation observed in cells of GD patients is coupled to changes in the chromatin structure to allow the silencing of gene expression.

## Non-Coding RNAs in AITD

A growing body of evidence suggests impaired expression of non-coding RNAs, and particularly of microRNAs (miRNAs) in autoimmune diseases (33). MiRNAs are small RNA molecules ranging from 18 to 25 nucleotides in length that bind to the 3′ untranslated region of target mRNAs and mediate their posttranscriptional regulation, leading to either degradation or translational inhibition, depending on the degree of sequence complementarity (37). MiRNAs target about 60% of all genes, and interact with other epigenetic mechanisms, such as DNA methylation and histone tail modifications, to organize the whole gene expression profile (38). Early studies in the field revealed several miRNAs that were differently expressed in cells from patients with AITD than in cells from healthy subjects (Table 1). For example, it was observed that the expression of miR-154*, miR-376b, and miR-431* was suppressed in PBMC from initial GD patients with respect to healthy controls, but recovered in GD patients in remission (39). Others observed that serum levels of miR-22, miR-375, and miR-451 were increased in patients with HT compared with healthy subjects and that serum levels of miR-16, miR-22, miR-375, and miR-451 were increased in patients with GD (40), while another study revealed significant variations of miR-200a and miR-155 in purified CD4+ T-cells and CD8+ T-cells of patients suffering from GD and HT (41). More recent studies attempted to explain the biological significance of miRNA deregulation or their possible clinical implications in AITD (42–46). For example, it has been proposed that increased miR-155 and decreased miR-146a may promote ocular inflammation and proliferation in Graves’ ophthalmopathy (42) and that circulating levels of miR-146a and interleukin 17 are significantly correlated with the clinical activity of Graves’ ophthalmopathy (43). It was also observed that miR-346 regulates CD4(+)CXCR5(+) T cells by targeting Bcl-6, a positive regulator of follicular helper T cells, and might play an important role in the pathogenesis of GD (44). Similarly, a decreased expression of miR-125a-3p was shown to upregulate interleukin-23 receptor levels in patients with HT (45). Increased expression levels of the miRNA let-7e were observed in PBMC of HT patients compared with those in GD patients and healthy volunteers, and it was shown that let-7e may be associated with the pathogenesis of HT through the regulation of intracellular interleukin 10 expression (46).

Limited data are available concerning miRNA expression in the thyroid gland of AITD patients. In this regard, miR-142-5p, miR-142-3p, and miR-146a showed high expression in HT thyroid gland (47). Furthermore, miR-142-5p was also detected in HT patient serum and positively correlated with thyroglobulin antibody (47). In addition, the overexpression of miR-142-5p in HT thyrocytes resulted in reduced claudin-1 mRNA and protein levels (47). Claudin proteins are major constituents of the tight junction complexes that regulate the permeability of epithelia, and miR-142-5p-mediated reduced expression of claudin-1 led to an increased permeability of thyrocytes monolayer (47). Another study showed a differential expression of 23 miRNAs in thyroid tissue of GD patients, resulting in the upregulation of 1,271 mRNAs and in downregulated expression of 777 mRNAs (48). Particularly, an integrated analysis of differentially expressed miRNAs and their target mRNAs demonstrated that miR-22 and miR-183 were increased in thyroid tissue of GD patients while their potential target mRNAs were decreased. On the contrary, miR-101, miR-197, and miR-6 were decreased while their potential target mRNAs were increased (48).

Indirect evidence of a possible involvement of miRNAs in AITD pathogenesis came also from studies linking polymorphisms in miRNA genes to increased AITD risk (49–51), so that there is increasing interest to clarify the variability in miRNA expression in order to better discriminate between miRNAs that are deregulated in a given disease, from others that could account for several autoimmune disorders (52, 53). In this regard, a deeper understanding of miRNA mediated networks in autoimmune diseases and their crosstalk with other epigenetic mechanisms that regulate gene expression levels is fundamental to elucidate the potential translational implications of these biomarkers (52, 53). In addition, there is increasing evidence that other non-coding RNAs than miRNAs, such as for example long non-coding RNAs, might play a role in autoimmune diseases, even if evidence in AITD is still limited (54).

## Concluding Remarks

Autoimmune thyroid disease patients can be clinically categorized into those with hyperthyroidism (GD), those with hypothyroidism (HT), and euthyroid subjects harboring thyroid autoantibodies (7). However, despite their phenotypic differences, it is believed that AITD patients share some common etiological factors (7), and genetic studies have revealed that if certain genes are unique for GD or HT, others are common to both disorders or to AITD and other autoimmune diseases (10). Indeed, different AITD phenotypes are often seen in members of the same family (7), and a significant increase in the prevalence of certain other autoimmune disorders has been reported in AITD patients (9). Epigenetic changes have been observed in multiple autoimmune diseases, they can be induced by environmental factors, and are increasingly recognized as one of the mechanisms by which environmental factors can trigger autoimmunity (10, 11). In this regard, there is increasing interest in searching for epigenetically deregulated pathways that might be common to different autoimmune disorders, and others that characterize a given disease and might be relevant in the clinical setting for diagnostic, prognostic, and therapeutic purposes (5). For what is concerning AITD there is increasing evidence of epigenetic changes in these conditions, but the available studies are still limited (Table 1) to be translated into the clinical settings. Particularly, the two available genome-wide DNA methylation studies in blood AITD cells are limited to GD patients (31, 32), and one of them included only three patients and three matched controls (27) making it difficult to clearly discriminate disease-specific epigenetic changes from others that could result from interindividual variability. Also data concerning histone tail modifications are mainly available from GD patients (32, 36), and lack of similar epigenome-wide data in cells from HT individuals does not allow comparing the two conditions in terms of epigenetic differences or similarities, so that the diagnostic values of the observed epigenetic changes and their potential prognostic utility are not yet clearly defined. Furthermore, methylation data in thyroid cells of AITD patients are limited to the study of a single gene (33). Data concerning miRNA expression in cells and tissues from AITD patients have been largely descriptive, and even if some investigators attempted to evaluate their potential clinical utility (42–46), data are still limited to be translated into the clinics. Epigenetic data are also lacking for another AITD, the postpartum thyroiditis, in contrast with postpartum psychosis, concerning which a study on miRNA expression was carried out (55). In this study, changes in miR-146a and miR-212 expression were observed in the 20 recruited patients with postpartum psychosis, but only 3 patients developed autoimmune thyroiditis, the small number impeding statistical analysis (55).

In addition, at best of my knowledge, data linking environmental exposures to specific epigenetic changes in AITD as well as studies evaluating the crosstalk between different epigenetic mechanisms are largely missing.

In conclusion, many investigators observed epigenetic changes in cells from AITD patients, but additional studies are required to confirm the observed changes and relate them to altered pathways that could be peculiar of a certain disease or of a certain environmental exposure, as well as to clarify common pathways in autoimmunity that could justify the onset of different autoimmune phenotypes in related family members, or in the same individual, in relation to different environmental exposures. Therefore, further research in this field could lead to a better understanding of the networks involved in disease pathogenesis, thereby opening the way for potential diagnostic and prognostic tools, as well as for epigenetic interventions in the patients based on miRNA silencing and/or chromatin remodeling agents.

## Author Contributions

The author confirms being the sole contributor of this work and approved it for publication.

## Conflict of Interest Statement

The author declares that the research was conducted in the absence of any commercial or financial relationships that could be construed as a potential conflict of interest. The handling editor declared a shared affiliation, though no other collaboration, with the author and states that the process nevertheless met the standards of a fair and objective review.
